# Neurodiversity and Autism Intervention: Reconciling Perspectives Through a Naturalistic Developmental Behavioral Intervention Framework

**DOI:** 10.1007/s10803-021-05316-x

**Published:** 2021-10-13

**Authors:** Rachel K. Schuck, Daina M. Tagavi, Kaitlynn M. P. Baiden, Patrick Dwyer, Zachary J. Williams, Anthony Osuna, Emily F. Ferguson, Maria Jimenez Muñoz, Samantha K. Poyser, Joy F. Johnson, Ty W. Vernon

**Affiliations:** 1 Gevirtz Graduate School of Education, University of California, Santa Barbara, Santa Barbara, CA USA; 2 Department of Psychology, University of California, Davis, Davis, CA USA; 3 Center for Mind and Brain, University of California, Davis, Davis, CA USA; 4 Medical Scientist Training Program, Vanderbilt University School of Medicine, Nashville, TN USA; 5 Department of Hearing and Speech Sciences, Vanderbilt University Medical Center, Nashville, TN USA; 6 Vanderbilt Brain Institute, Vanderbilt University, Nashville, TN USA; 7 Frist Center for Autism and Innovation, Vanderbilt University, Nashville, TN USA; 8 Spectrum Support, LLC, Baltimore, MD USA

**Keywords:** Autism, Neurodiversity, Naturalistic developmental behavioral intervention

## Abstract

Proponents of autism intervention and those of the neurodiversity movement often appear at odds, the former advocating for intensive treatments and the latter arguing that autism must be accepted as a form of diversity. The history of behavioral intervention has understandably outraged many in the Autistic community, though many still value supports focused on quality of life. This commentary argues that Naturalistic Developmental Behavioral Interventions (NDBIs) hold promise for bridging the gap between early intervention and the neurodiversity movement. However, we recognize NDBIs have much room to grow and suggest multiple strategies for improvement. We believe these updates are not only feasible for clinicians and researchers to implement but will ultimately lead to improved quality of life for Autistic individuals.

At first glance, the perspectives and objectives of autism intervention proponents and neurodiversity advocates appear contradictory. The former advocate for treatments that can help establish and improve foundational social communication skills, interpersonal competencies, behavioral flexibility, and self-regulation strategies. They point to evidence in the research literature suggesting that without early and ongoing intervention, there are significant developmental consequences that can ultimately limit opportunities, the likelihood of desirable personal outcomes, and associated quality of life for individuals on the spectrum (see Fuller & Kaiser, 2019, and Landa, 2018 for a recent review and meta-analysis). Proponents of the neurodiversity movement, on the other hand, maintain that autism (and other forms of neurocognitive differences like ADHD and dyslexia) are simply dimensions of the rich diversity of the human experience, characterized by a different means of experiencing, processing, understanding, and interacting with the world (Chapman, 2019). While there is important variability in the perspectives of neurodiversity advocates—as the neurodiversity movement is decentralized and continuously evolving through dialogue (Chapman, 2020; Singer, 2020)—there is a general consensus that the unique attributes associated with autism and other neurocognitive disabilities should not be distilled down to a set of symptoms and vulnerabilities in need of correction. Instead, neurodiversity perspectives assert a need to promote societal education, acceptance, and accommodation of the neurocognitive differences of these individuals. In recent years, there has been a widening division between these two groups, with efforts to reconcile or mediate differences often ending in hostile exchanges or stalemates.

The purpose of this commentary is to identify and explore areas of overlap between the values and objectives of the neurodiversity movement and contemporary autism intervention approaches, specifically in the context of Naturalistic Developmental Behavioral Interventions (NDBIs; Schreibman et al., 2015). This work was intentionally crafted through a collaboration of Autistic^1^ and non-Autistic co-authors to ensure that important perspectives and discussions were captured and synthesized. Further, we hope to demonstrate and encourage a collaborative effort between these two groups as a model for future partnership.

Due to the controversial nature of this topic, it is important to highlight our positionality when considering the views presented in this paper. Three authors identify as Autistic (PD, ZJW, JFJ). At the time this manuscript was initially drafted, nine of the authors were doctoral students in the following disciplines: education (RKS, KMPB, SKP), clinical psychology (DMT, AO, EFF, MJM), developmental psychology (PD), and psychiatry/neuroscience (ZJW). All except two students (PD, ZJW) have experience as NDBI clinicians at an autism center where TWV is a faculty member and center director (though ZJW was trained in NDBI implementation during a summer program at the center). Two doctoral students (KMPB, MJM) are Board Certified Behavior Analysts (BCBAs), as are TWV and JFJ. KMPB and JFJ both work for community-based applied behavior analysis (ABA) companies providing behavioral intervention to Autistic individuals. In her clinical practice, JFJ does not implement NDBIs specifically, though she uses many of the same naturalistic, client-led principles espoused by NDBIs. She also runs a neurodiversity consulting business and an Instagram page focused on being #ActuallyAutistic and reforming behavioral intervention. Though specific author research interests vary within the field of autism (e.g. social validity of intervention/education, implementation and dissemination of interventions, sensory processing and attention, outcome measurement, etc.), all authors are committed to improving interventions using a pro-neurodiversity framework.

This commentary begins by discussing the models of disability that underpin both perspectives. Next, we provide a brief historical context of behavioral interventions, including their controversial past and their gradual evolution into more naturalistic approaches. In parallel, we also explore the historical origin, evolution, and contemporary perspectives of the neurodiversity movement. We then identify NDBIs as a promising framework that has the potential to bridge autism intervention and neurodiversity tenets based on overlap in values, perspectives, and ultimate goals. Lastly, suggestions to improve alignment between NDBIs and the neurodiversity movement are discussed.

We intend to question the assumption that contemporary behavioral interventions necessarily run counter to the goals of the neurodiversity movement and challenge the widely-held belief that all behavioral interventions are inherently harmful to Autistic people (see Chapman & Bovell, 2020 for further discussion). On the contrary, we argue that fostering a collaborative partnership between perspectives can be mutually beneficial for furthering the aims of both parties. Specifically, naturalistic intervention approaches can center Autistic perspectives, become even more attuned to the needs and personal goals of the recipient, and focus on co-constructed strategies and goals. As such, these person-centered interventions can empower Autistic individuals, equip them to set and accomplish personally meaningful goals, and ultimately improve their overall quality of life.

## Models of Disability

It is important to acknowledge that the lens through which we view and interpret challenges and vulnerabilities is inextricably anchored to our underlying conceptualization of autism and disability more generally. Historically, many therapies and treatments targeting challenges associated with autism were borne out of the long-dominant medical (or pathological) model of disability (Hogan, 2019). The medical model views diagnoses such as autism through the lens of associated deficits or impairments, which is encapsulated in the very name of the condition, Autism Spectrum *Disorder* (American Psychiatric Association, 2013). Under the medical model, clinical impairment is an essential prerequisite for diagnosis, with such impairments recognized as prime intervention targets to be remedied by clinical treatment. The medical model focuses on vulnerabilities and deficits grounded in a disease and disorder-oriented framework, without necessarily considering what that person needs to function and thrive (Baker, 2011).

Early behavioral treatments stemming from the medical model did not consider issues of neurodiversity, social validity, and person-centered approaches to care. Over the last several decades, however, there has been growing recognition of the need for therapeutic approaches that not only consider the strengths, preferences, goals, and values of individuals on the spectrum and their families, but also allow for co-construction of therapy based on the respective expertise of Autistic individuals, family members, and clinicians (Gabovitch & Curtin, 2009). Despite these advancements, more work remains to be done as long as clinicians continue to prioritize correcting perceived deficits as the ultimate goal of behavioral intervention.

One alternative to the medical model of disability is the social model, under which *society* is understood to give rise to disabled people’s challenges and disabilities and thus, bears responsibility for their struggles (Oliver, 1990). Within the strict social model of disability, while individual variations may be linked to impairment, an individual’s impairments do not lead to experienced disability unless society fails to acknowledge, include, and accommodate them.

However, many neurodiversity advocates follow Singer, who originally coined the term “neurodiversity,” in rejecting strict versions of both social and medical models of disability (Singer, 1998). They instead suggest that Autistic people’s challenges are caused by a poor fit between the individual characteristics of Autistic people and the unaccommodating norms and expectations of the sociocultural context in which they are situated (Bailin, 2019; Ballou, 2018; Kapp, 2013; Singer, 2019), with neither society nor the individual necessarily being the sole cause of disability (see also Lai et al., 2020). Different variants on this sort of interactionist approach to disability exist, such as Scandinavian interactionist models of disability (see Gustavsson, 2004; Tøssebro, 2004), social-relational approaches (see Reindal, 2008; Thomas, 2004), and Chapman’s (2021) social-ecological model, but they hold in common the implication that remedial efforts can take place on both individual and institutional levels.

## History of Autism Intervention

Research aimed at improving the lives of Autistic individuals has been conducted for decades. Interventions have attempted to ameliorate various challenges often associated with autism, with many interventions focused on the promotion of language, communication, socialization, independent living, and emotion regulation skills. However, the history of this research has not been without its share of controversy and misguided efforts. This history plays a prominent role in the way researchers and clinicians approach autism interventions today (see Donvan & Zucker, 2016; Feinstein, 2010; Grinker, 2007; Nadesan, 2013; Silberman, 2015).

As cultural and societal norms shifted in the United States throughout the mid-to-late twentieth century, so did the views of autism and autism intervention. The 1940s saw the gradual introduction of autism as its own separate diagnostic category. Children who were previously seen as “psychotic” (indeed, distinguishing autism from childhood schizophrenia took time; see DeMyer et al., 1971; Kanner, 1949) or “retarded” came to be viewed as “highly intelligent but with a tendency towards social withdrawal and emotional limitations” (Kanner, 1943, p. 220). Perhaps in reaction to eugenicist approaches to disability (Feinstein, 2010), the psychiatry of the time failed to acknowledge many of the hereditary, genetic, and environmental factors now known to be associated with autism. Instead, many professionals during the 1950s asserted that autism was caused by “Refrigerator Mothers” who lacked the requisite levels of interpersonal warmth and maternal caring toward their children (Cohmer, 2014; Donvan & Zucker, 2016; Feinstein, 2010; Pollak, 1997; see also Kanner, 1949). As a result, a commonly used treatment method was to remove children from their families and institutionalize them.

The 1960s and 70s brought about the emergence of behavior therapy for children with autism (Ferster & DeMyer, 1962; Lovaas et al., 1974; Mazuryk et al., 1978). These early intervention programs incorporated both reinforcement and punishment techniques to drive behavior change in areas such as social skills, communication, and disruptive behavior (Dixon et al., 2012). Use of aversives was common at this time, including the use of electric shocks, physical slaps, and yelling (Moser & Grant, 1965). Researchers and interventionists readily conceded that shocks and other aversives provoked fear and distress in children, but justified these practices by arguing that they generally yielded social responses and other positive effects (Lichstein & Schreibman, 1976; Rechter & Vrablic, 1974; Simmons & Lovaas, 1969; Tate & Baroff, 1966). Furthermore, the use of aversives was purported to be associated with greater feelings of personal accomplishment for professionals (Harris et al., 1991). (Although it is rightfully no longer widespread or widely accepted today, aversive electric shock nevertheless continues to be used at the Judge Rotenberg Center, despite outcry from advocates and ongoing legal battles to ban its use (Autistic Self Advocacy Network, 2021; McFadden et al., 2021).)

The emergence of behavior therapy in the 1970s paralleled a major shift in etiological understanding of autism and commonly accepted interventions (e.g., Rimland, 1964). In the 1960s and 1970s, many parents founded advocacy organizations and worked to resist the attempts of researchers and professionals to blame them for their children’s autism diagnosis (Donvan & Zucker, 2016; Feinstein, 2010). These efforts were reinforced in the 1970s by findings from emerging twin study methodologies, studies of maternal rubella, and advances in genetics (Chess, 1971; Folstein & Rutter, 1977; Gillberg & Wahlström, 1985), leading the field to view autism as a biological condition. Indeed, the field of psychiatry as a whole saw an increased use of psychotropic medications for behavioral disorders at this time (King & Bostic, 2006). Autism was no longer blamed on the individual or their family and was instead recognized as part of biology. As the field embraced this biological understanding, many parents began administering drugs and vitamins to their children in hopes of reducing autism traits or even “curing” their child’s autism (Rimland, 1974, 1988). These drugs and supplements were followed by a long series of pseudoscientific, and sometimes dangerous, biomedical autism treatments (Offit, 2008) that continue in some form to the present day (Höfer et al., 2017).

Meanwhile, in the late 1980s, behavior intervention underwent a shift that would shape the field for the next three decades. In 1987, psychologist Ivar Lovaas published a study that concluded that 47% of Autistic children who received 40 h a week of intensive behavioral therapy would go on to demonstrate cognitive abilities in the normal range and attain mainstream educational placements, in comparison to 2% of control children (Lovaas, 1987). Though this study and Lovaas’s approach to behavioral intervention have been widely criticized in the decades since (e.g. Gibson & Douglas, 2018; Kirkham, 2017), this investigation was the beginning of the now widespread field of applied behavior analysis (ABA), which remains one of the most widely used interventions for skill acquisition and reduction of harmful behavior for Autistic individuals today (Becerra et al., 2017; Xu et al., 2019). While this traditional form of ABA therapy (commonly referred to as Discrete Trial Training [DTT; Smith, 2001]) has been shown to have some beneficial effects on outcomes such as language, social functioning, adaptive behavior, cognitive ability, and challenging behaviors (Sandbank et al., 2020), there is a paucity of experimental evidence regarding long-term effects on adult outcomes (Jónsdóttir et al., 2018). Moreover, the literature has identified issues with some aspects of DTT’s implementation and outcomes, including slow progress, some practitioners’ continued use of aversives, and the potential lack of generalization and flexible application of skills in naturalistic contexts (Kirkham, 2017; Koegel et al., 1998; Sandoval-Norton & Shkedy, 2019; Schreibman, 2005).

At the same time during the 1980s, amidst the disability rights movement in the United States, there was a strong push for the inclusion of individuals with disabilities in educational and community settings. Modifications to interventions were thus necessary to support these individuals across environments. This helped pave the way for the next generation of intervention models that focused on social validity, naturalistic settings and routines, parent and stakeholder involvement, and positive strategies for behavior change and skill acquisition: Naturalistic Developmental Behavioral Interventions (Schreibman et al., 2015). NDBIs were developed to combat the rigidity and tediousness of traditional ABA and share core characteristics that are less medicalized, stigmatizing, and depersonalizing, and approach skill acquisition in a more accepting, compassionate, and empathetic manner. NDBIs enhance the components of traditionally-practiced ABA by creating a more motivational, naturalistic, comprehensive, and person-centered intervention.

## History of the Neurodiversity Movement

The neurodiversity movement arose out of the Autistic advocacy movement that, after emerging in the 1990s, began advocating for Autistic rights and societal acceptance of autism in response to the marginalization of Autistic people by professionals and organizations run by the parents of Autistic individuals (Chamak, 2008; Kapp, 2020; Pripas-Kapit, 2020; Sinclair, 2005, 2010). These established parent organizations approached autism using the medical model and supported treatments that sought to prevent, treat, and cure autism (Baker, 2011), with some treatments characterizing autism as an adverse experience distinct from the individual (Langan, 2011). Differing perspectives on “curing” autism have divided the community, with parents being more likely to endorse wanting a cure than Autistic individuals themselves and parent-led advocacy groups more likely to align with cure narratives (Carey et al., 2019; Kapp et al., 2013). However, it should also be noted that many parents have embraced the neurodiversity movement (Greenburg & Des Roches Rosa, 2020; Russell, 2020; Savarese et al., 2010).

The neurodiversity movement primarily differs from the Autistic advocacy movement in its greater breadth, extending beyond autism to capture multiple domains of neurodevelopmental difference. Broadly speaking, the neurodiversity approach defines atypical neurodevelopment (such as autism) as variation in the human experience that should be accepted and respected (Griffin & Pollak, 2009). Singer (1998) and Blume (1998) introduced the idea of neurodiversity into academia and popular media using the analogy of biodiversity; just as biodiversity is essential and necessary for healthy ecosystems, so too might neurodiversity contribute to human flourishing. Within this paradigm, an individual’s ability to contribute to society is not a prerequisite for acceptance; instead, advocates of neurodiversity understand acceptance itself as necessary for human dignity and well-being (Bailin, 2019; Ballou, 2018). Thus, this framework represents Autistic individuals as simply different as opposed to pathological (Broderick, & Ne’eman, 2008). Within this framework, the Autistic way of socializing, communicating, and sensing is seen as an alternate and acceptable form of human biology that should be celebrated and accommodated rather than corrected or cured (Davis & Crompton, 2021; Ortega, 2009).

## Changing Perspectives on Autism Intervention

Although the neurodiversity movement has advocated for a shift away from the medical model’s foundation for autism intervention, many neurodiversity advocates are still in favor of intervention when it is (a) provided in a respectful manner, (b) focused on teaching useful skills, and (c) improves subjective quality of life (Chapman & Bovell, 2020; den Houting, 2018; Kapp et al., 2013). Indeed, many individuals on the spectrum face personal challenges related to their experience as being Autistic, with many reporting that this difference makes them feel lonely and socially isolated (Ruiz Calzada et al., 2012). Robertson (2009) asserts that many Autistic individuals possess challenges in areas such as self-determination, social inclusion, well-being (material, emotional, and physical), personal development, interpersonal relationships, and rights. These domains could be targeted through personalized, strength-based interventions, which in turn, could lead to increases in overall quality of life.

However, there has been considerable criticism of contemporary behavioral interventions (e.g., Chapman & Bovell, 2020; Dawson, 2004; Wilkenfield & McCarthy, 2020), with Autistic individuals and neurodiversity advocates sometimes describing them as traumatic, particularly with regards to use of aversives and the suppression of Autistic traits/regulatory mechanisms (Bascom, 2015; Cumming et al., 2020; Gardner, 2017; Kupferstein, 2018; Sequenzia, 2016; Stop ABA, Support Autistics, 2019). Unfortunately, adverse events are seldom considered in research on non-pharmacological autism interventions (Bottema-Beutel et al., 2021a; Dawson & Fletcher-Watson, 2021), making it difficult to assess even immediate harmful impacts of ABA. Nevertheless, some researchers and individuals argue that ABA can decrease intrinsic motivation, self-confidence, and self-esteem due to its emphasis on compliance (Sandoval-Norton & Shkedy, 2019; Stop ABA, Support Autistics, 2019), and some assert that all intervention based on behavioral theory is flawed, given its typical lack of acknowledgement of mental states (Therapist Neurodiversity Collective, n.d.; Tolley, n.d.). Researchers have also drawn attention to the abundance of conflicts of interest in ABA research, which are seldom fully disclosed (Bottema-Beutel et al., 2021b). Thus, although advocates generally want to provide adequate support in order to improve quality of life, the sharply negative experiences of many Autistic people have fostered strong opposition to these interventions in many quarters, to the extent that discussion of ABA is considered triggering or forbidden in some online communities (e.g., Ask me, I’m Autistic (24 h rule!), n.d.). On the other hand, *opposition* to ABA is marginalized or banned in some online professional communities (e.g., ABA Skill Share, n.d.). This limits opportunities for dialogue and clarification of misconceptions between these competing viewpoints, leading to sometimes heated debates on online forums (see comments on a *Spectrum News* article regarding ABA controversies; Devita-Raeburn, 2016). With limited communication between neurodiversity advocates and providers/consumers of ABA, behavioral intervention is likely to carry on as is, without fully addressing Autistic individuals’ concerns. Moreover, maintenance of this status quo runs the risk of further perpetuating the perception of ABA as both abusive and incapable of improvement. This unfortunate state of affairs is unlikely to improve without meaningful collaboration between both groups, and by convening a diverse group of Autistic and non-Autistic stakeholders to discuss these controversial topics and learn from each other, we collectively aim to bridge the gap between opposing viewpoints and improve the alignment of behavioral intervention with the tenets of neurodiversity. However, we also recognize that individuals in both the clinical and autism communities will likely disagree with some of our claims and suggestions, which will hopefully spark further fruitful conversations and reform efforts.

Ultimately, we believe that behavioral intervention can be compatible with the neurodiversity paradigm, although substantial reform is needed to adequately incorporate the tenets of neurodiversity into behavioral intervention theory and practice, as has been argued by several other researchers. For example, Fletcher-Watson (2018) argues that it is possible for early interventions to fit within a neurodiversity framework if they utilize strength-based support and measurement, are modeled on Autistic learning and play, have Autistic-led targets and outcome measures, and utilize the natural environment. Similarly, Lai et al. (2020) call on intervention providers to work together with families and individuals to minimize barriers, maximize potential, and increase person-environment fit. Leadbitter et al. (2021) recently suggested that in order to engage with neurodiversity, interventions must redefine effectiveness, lead to outcomes endorsed by the Autistic community, and rely on partnerships with Autistic individuals. We agree with the stances laid out by these researchers and extend their arguments to show how NDBIs, while already much more closely aligned with these ideals than other behavioral approaches, can be further improved upon in order to fully integrate with an interactionist neurodiversity perspective.

## Theoretical Alignment of NDBIs with Neurodiversity

Overall, behavioral intervention for Autistic individuals has become increasingly naturalistic and person-centered, with even more traditional forms of ABA making progress in this regard (Leaf et al., 2017). NDBIs (Schreibman et al., 2015) have emerged as a group of particularly promising naturalistic interventions that have been shown to have positive effects on standardized measures of language and social-communication (e.g. Sandbank et al., 2020; Tiede & Walton, 2019). A recent meta-analysis by Sandbank et al. (2020) demonstrated that, for children up to 8 years old, NDBIs yielded more favorable developmental outcomes when compared to other interventions. While a recent direct comparison of an NDBI with traditional DTT found no between-intervention differences in child outcomes such as social communication and cognitive ability (Rogers et al., 2020), studies have yet to compare NDBIs and DTT in their effects on autistic people’s quality of life, perspectives on intervention social validity, and reported psychological well-being.

All models included under the NDBI umbrella share common features and components, which are informed by both behavioral and developmental theory. Though NDBIs incorporate the behavioral principle of positive reinforcement (in which someone will likely repeat a behavior that led to positive consequences), their basis in developmental theory ensures that individuals are not viewed simply as a pattern of behavioral antecedents and responses. Instead, personal preferences, opinions, motivations, and social relationships are acknowledged and appreciated. NDBI components include implementation in natural settings, shared control between the child and the teaching partner, utilization of natural contingencies, and use of behavioral strategies to teach developmentally appropriate skills (Schreibman et al., 2015; Vivanti & Zhong, 2020). They also employ individualized treatment goals, focus on child-initiated teaching episodes, capitalize on natural reinforcement and child motivation, and can include adult imitation of the child. While some of these aspects may be present in modern-day, progressive forms of DTT (Leaf et al., 2017), these naturalistic components are the explicit procedural components of NDBIs. Examples of some of the most researched of these interventions are Pivotal Response Treatment (PRT; Koegel et al., 2016), the Early Start Denver Model (ESDM; Rogers & Dawson, 2020), Joint Attention, Symbolic Play, Engagement, and Regulation (JASPER; Kasari et al., 2006), Incidental Teaching (McGee, 2005), and Project Improving Parents as Communication Teachers (Project ImPACT; Ingersoll & Wainer, 2013). Multiple randomized-controlled trials with young children suggest NDBIs may be particularly effective in supporting the development of early social communication, language, and play skills (e.g. Dawson et al., 2010; Gengoux et al., 2019; Ingersoll et al., 2016; Vernon et al., 2019).

NDBIs can be a great starting place for clinicians and researchers who strive to provide an intervention for young Autistic children that highlights strengths and prioritizes individual preferences. These naturalistic, play-based interventions possess several of the characteristics outlined by Fletcher-Watson (2018). Alignment between NDBIs and neurodiversity is best captured by their focus on stakeholder agency within the intervention, their strength-based approach, and their implementation in the natural environment.

### Co-construction and Agency in Intervention Experience

The components of NDBI models intentionally alter the hierarchical power structure that traditionally exists between adult (clinician or parent) and child in a treatment context (Schreibman et al., 2015). Rather than adults dictating the session structure and children serving as passive recipients of a pre-determined therapeutic protocol, the children are viewed as active participants in a more constructivist, child-led approach. Child preferences and sustained motivation are core considerations, highlighted through their dynamic selection of the materials and activities used in an NDBI session (Minjarez & Bruinsma, 2020; Vivanti & Zhong, 2020). Child initiative and spontaneous communication attempts are encouraged and fostered. The adult role is to build upon and foster existing child motivation to engage and communicate, rather than imposing a therapeutic agenda that might run counter to the child’s interests and desires. This allows children to learn through experiences that are personally relevant and meaningful to them, while also motivating (rather than repetitively drilling) them to acquire functional skills. The focus on child co-construction of therapy fosters a more equitable learning environment while simultaneously protecting against ‘norm-driven’ interventions focused on symptom reduction (as an end in and of itself) and behavioral compliance.

Strong parent partnerships also ensure that the family has agency in the intervention experience. This collaborative relationship is a core tenet of NDBIs and is accomplished by actively including family members in the decision-making process, facilitating caregivers as intervention providers, and accounting for child preferences. Intervention goals within the NDBI context must be culturally acceptable to families and focus on functional skills that can be improved upon within a family’s established routine (Gengoux et al., 2020). While all NDBIs can be delivered by clinicians, parent education is emphasized and leads to increased intervention exposure and increased opportunities for naturalistic learning (Schreibman et al., 2015). Educating and training parents to fidelity can also lead to an increased sense of empowerment and confidence within parents (Minjarez et al., 2020), which can in turn lead to improvements in child engagement (Brookman-Frazee, 2004).

### Strength-Based Approach

NDBIs generally follow a strength-based approach and use child motivation as a core asset in treatment. Within these models, clinicians take advantage of a child’s existing interests and preferred activities as a means to maximize engagement, responsiveness, and skill acquisition. These models use existent prerequisite skills to further develop skills that are developmentally and personally appropriate to the child. For example, a child who cannot fully say the name of a preferred social activity will still be granted access to the social interaction in response to an attempted verbal request. This strategy allows for tailoring of intervention goals based on individual profiles and parent input, rather than imposing a set of intervention goals determined solely by a clinician (Gengoux et al., 2020). Additionally, with their emphasis on child strengths and preferences, NDBIs do not focus on behavior reduction. Because of this, the unique traits of Autistic individuals can be celebrated and incorporated into teaching skills most relevant to them—skills that they will be able to use to further explore their preferred interests and strengths.

### Emphasis on Naturalistic Skill Building

While more progressive forms of DTT strive for naturalistic teaching environments (Leaf et al., 2017), traditionally-practiced ABA continues to emphasize seated table activities directed by the clinician (Bogin et al., 2010; Leaf et al., 2017). In contrast, NDBIs should be conducted in natural play and learning contexts within the child’s everyday environment (Schreibman et al., 2015; Vivanti & Zhong, 2020). As such, NDBIs do not need to be implemented in the context of an “intervention session” (though they could be); instead, opportunities to use NDBI principles can be interspersed throughout a child’s daily routine, whenever the occasion naturally arises (with adults making sure to not overburden children with an excessive amount of skill practice). For example, a child’s desire to play outside could be used as an opportunity to teach new language concepts. Additionally, all reinforcement within the context of NDBIs is also *natural—*that is, the reinforcer is simply the natural result of whatever the child did (Schreibman et al., 2015; Vivanti & Zhong, 2020), mirroring the same cause-and-effect relationships inherent in non-intervention contexts. For example, if a child asks for a preferred item, the natural reinforcer is the preferred item. If a child is prompted to discuss a preferred interest with a peer who has the same interest, the conversational response (focused on their interest) might act as a natural reinforcer, with the child learning that new knowledge and joint discussion of a favorite thematic interest is possible when discussing that interest in a conversation with others. Here, there is no explicit goal to normalize behavior or make the child more neurotypical. Instead, the child is motivated to build functional social and communicative skills in an enjoyable, naturally reinforcing conversational exchange. In fact, if this conversation were *not* naturally reinforcing to the child, encouraging the child to participate in the conversation would not be considered “good NDBI implementation.” Ideally, in all NDBI teaching opportunities, the child’s interest is honored, and the reinforcement parallels the natural, logical consequences of interaction that would occur for any other individual. By employing this kind of naturalistic reinforcement, children can learn meaningful skills in an enjoyable and meaningful way, allowing them to better achieve their self-defined goals in everyday situations.

As such, NDBI models can allow Autistic individuals to interact with the world around them in a way that aligns with their individual preferences and cognitive functions, which ultimately improves their quality of life by increasing agency and autonomy. These child-led and naturalistic approaches align with the neurodiversity approach’s emphasis on personal strengths and self-determination. They encourage clinicians and parents to let children “teach you a little of her language, guide you a little way into his world,” as Sinclair (1993) advised in *Don’t Mourn For Us*. However, as neurodiversity ideals stem from the experiences of Autistic individuals, it is imperative that professionals and researchers honor these perspectives when creating, monitoring, and improving intervention, as these individuals are the most important stakeholders in this iterative process. Though we have outlined the strengths that NDBIs hold in relation to neurodiversity, there are still many areas where NDBIs (particularly as they are currently practiced) must continue to evolve in order to truly celebrate neurodiversity and benefit Autistic children in the short and long term.

## Improving the Alignment of NDBIs with Neurodiversity

One of the most important contributions of the neurodiversity movement is the idea that Autistic behaviors, interests, challenges, and strengths all represent valid ways of being. A key criticism leveled at ABA (including NDBIs; see Des Roches Rosa, 2020) is that treatment goals often reflect a neurotypical interpretation of ideal or normative human behavior, as opposed to being focused on personal, Autistic-centered ways of thinking and acting (Michael, 2018; Neurodivergent K, 2013; Ludwig, n.d.). Even autistic individuals who have had positive experiences with behavioral intervention highlight that it can at times be too preoccupied with making autistic children appear “normal” (Lamb, 2019; Lowery, 2017). In his “seminal” paper, Lovaas (1987) contended that his behavioral intervention led to “recovery” from autism, with those who had been previously diagnosed now “indistinguishable from their normal friends” (p. 8). The underlying message, of course, was that elimination of autism “symptomatology” was the ultimate objective. “Recovery” and “normalcy,” however, are not the goals that most Autistic individuals hope to achieve (Davison, 2018; Gillespie-Lynch et al., 2017; also see the 2015 anthology *Loud Hands* (Bascom, 2015) in which many Autistic advocates decry the notion that they need to “act neurotypical”). While respecting individuals’ needs and autonomy is already an integral part of NDBIs, clinicians must take care to recognize that autonomy goes deeper than simply allowing for child choice.

We recommend that clinicians and researchers use multiple strategies to continually improve upon the respectfulness and individualization of early intervention models including: soliciting input from older Autistic individuals, considering the social and ecological validity (i.e. perceived acceptability and usefulness by Autistic individuals), conducting participatory-based research when feasible, and using adaptive treatment designs to respond dynamically to participant needs. A summary of these recommendations can be found in Table 1. While our recommendations refer to NDBIs, whose principles provide a useful reference point for reforming behavioral intervention, our suggestions herein apply to all intervention providers, within the field of ABA and beyond.

**Table 1:** Summary of recommendations to improve alignment of NDBIs with the neurodiversity paradigm

Overall area	Description	Practical recommendations
Centering autistic voices	Clinicians and researchers should seek out input from Autistic individuals regarding intervention goals and implementation, as certain social and behavioral goals may be harmful to Autistic people’s well-being, as might pathology language and assumptions	Hire Autistic consultants Are the goals appropriate and respectful? Is the intensity appropriate? Is the implementation appropriate and respectful? Hire Autistic interventionists Provide psychoeducation related to neurodiversity to parents (in collaboration with Autistic consultants/employees) as early as the first diagnostic feedback session Encourage autism acceptance Direct attention to child strengths Suggest alternatives to pathology language
Improving social and ecological validity	Social validity must be assessed in all intervention contexts, particularly from the perspective of the Autistic client More needs to be known about the extent to which community NDBIs reflect the core values of both NDBIs and neurodiversity	Include social validity measures in all NDBI research trials For example, the STP (Berger et al., 2016) Include self-report measures if possible Include measures of Quality of Life to ensure correspondence between intervention and real-life outcomes Develop measures of social validity for non-speaking individuals Survey community providers Ensure measures of social validity are statistically validated to limit subjectivity Evaluate cultural biases and acknowledge cultural differences
Improved research design	Participatory action research Disclosure of conflicts of interest Reporting of adverse events Adaptive treatment designs	Include stakeholders, particularly Autistic ones, from the beginning of all research projects Intervention researchers must disclose any potential COI related to the intervention being investigated All behavioral intervention trials must assess adverse events, including psychological distress Allow for flexibility in research such that interventions can be individualized

### Autistic Perspectives on Intervention

Clinicians implementing NDBIs recognize that parents have enormous insight into their child’s strengths and weaknesses and make it a point to collaborate with family members when designing and implementing an intervention plan. Though neurodiversity advocates recognize the importance of parents in supporting the development of Autistic children (Kapp, 2018), when both parents and clinicians are not on the spectrum, the Autistic perspective is likely lacking. Research with Autistic adults has revealed that some take issue with certain social and behavioral intervention goals that are formulated without their input (Gillespie-Lynch et al., 2017), which are often misaligned with their personal values.

#### Social Features of Autism and Camouflaging

While NDBIs do not have the explicit goal of eradicating Autistic traits, reductions in autism symptom severity are often highlighted as an intervention outcome in published literature (e.g., Dawson et al., 2010; Vivanti & Zhong, 2020; Vivanti et al., 2019). While many of the skills targeted in these studies may lead to a positive effect on quality of life, the explicit focus on reducing autism “severity” implies that, all other things being equal, being less Autistic is something to aspire to. Moreover, intervention goals may focus on behavior patterns and social communication skills observed in neurotypical children, such as an emphasis on joint attention and imitation skills (Schreibman et al., 2015). While these may be acceptable and welcomed goals for some Autistic children, some Autistic people find “typical” social behavior such as eye contact to be thoroughly uncomfortable and distressing, even if they have learned how to tolerate or feign use of these behaviors (Trevisan et al., 2017).

Additionally, many Autistic adults report negative subjective outcomes as a result of having to meet inappropriate and unrealistic benchmarks, such as feeling they have to “pass” as neurotypical and mask or “camouflage” their Autistic behaviors (Bargiela et al., 2016; Cage & Troxell-Whitman, 2019; Cook et al., 2021; Hull et al., 2017; Livingston et al., 2019). In some cases, pursuit of these objectives has been cross-sectionally associated with poor mental health, reduced wellbeing, and suicidality (Cassidy et al., 2018; Hull et al., 2019, 2021), although causal relationships have yet to be demonstrated (Williams, 2021). Furthermore, first-person accounts of “Autistic burnout” warn of the cumulative load of excessive camouflaging and other stressors (Higgins et al., 2021; Raymaker et al., 2020), raising questions about traditional assumptions that high intervention intensity is always desirable (see also discussion by Pellecchia et al., 2019, and a recent randomized trial by Rogers et al., 2020 demonstrating limited effects of intervention intensity). Clinicians must therefore assess the value of intervention goals and tailor the dose and intensity to individual needs and experiences.

An excellent way for NDBI providers to ensure their approach does not pressure individuals to engage in camouflaging is to base intervention goals on Autistic, rather than neurotypical, behavior (Fletcher-Watson, 2018). Providers must examine goals and determine if they constitute functional adaptive skills and will ultimately improve quality of life (AStrangerinGodzone, 2011; Robertson, 2009). A closer look into the selected goals may reveal that they actually seem to focus on enhancing social mimicry (i.e. making the child appear more typical) without explicitly promoting well-being. It is imperative to keep in mind that even Autistic people who lack fluency in “neurotypical” social skills can engage in successful interactions and form meaningful, reciprocal social bonds with others on and off the spectrum (Chen et al., 2021; Crompton et al., 2020; Davis & Crompton, 2021; Heasman & Gillespie, 2019). It is inappropriate, ill-advised, and unethical to teach and encourage certain skills just because they are considered normal, as this may contribute to the internal/external incongruence experienced by many Autistic individuals that ultimately may impact their mental health. Therefore, it is prudent to ensure that Autistic children and adults have access to both neurotypical and Autistic peers so that they can develop awareness and understanding of the diverse definitions of socially acceptable behavior. Providers must also understand that social challenges do not lie solely within an Autistic person—they are *relational*, meaning that the interactional partner shares responsibility for preventing and remedying misunderstandings and social rejection (Davis & Crompton, 2021; Keating & Cook, 2020; Milton, 2012; Sasson et al., 2017). Indeed, Autistic people have argued that neurotypical people often have difficulty understanding and having empathy for Autistic people (Milton, 2012), a claim supported by the research literature (Alkhaldi et al., 2019; Edey et al., 2016).

#### Autistic Repetitive Behaviors and Special Interests

Repetitive behaviors and focused interests are important to the internal regulation and well-being of many Autistic individuals (Kapp et al., 2019; Manor-Binyamini & Schreiber-Divon, 2019; Milton & Sims, 2016; see also Grove et al., 2018). Even when such behaviors are not targeted explicitly in NDBIs, they are often still pathologized in both implicit and explicit ways, and clinicians still may establish a secondary goal of reducing or eliminating them (Leekam et al., 2011). Interventionists must decide whether or not behaviors are truly detrimental (e.g. when they are potentially or actually self-injurious, aggressive, or destructive) before attempting to extinguish them. Non-harmful self-stimulatory or repetitive behavior is often used to regulate emotions, and these behaviors may serve as adaptive coping skills for many individuals (Kapp et al., 2019; Manor-Binyamini & Schreiber-Divon, 2019). Moreover, the perceived stigma associated with repetitive behaviors arises primarily from societal norms (Kapp et al., 2019). Autistic individuals may also use repetitive behaviors and interests as a way of developing social connections and some report enjoying “interactive stimming” with others (Sinclair, 2005). If certain behaviors are truly dangerous, clinicians are encouraged to conduct a thorough functional assessment (Beavers et al., 2013) and determine methods for identifying and eliminating triggers while also teaching replacement behaviors that fulfil the same function (e.g. sensory input, task avoidance, emotion regulation, etc.).

### Improving Practices and Validity

#### Neurodiversity, Autism Acceptance, and Terminology

To further improve the alignment of autism intervention with neurodiversity, we recommend that interventionists maintain a dialectic synthesis between the goals of autism acceptance and the desire to facilitate the development of core functional skills—holding both perspectives in mind simultaneously and valuing both viewpoints without rejecting or diminishing the importance of either one. However, as this is not the current standard in the field, many intervention providers and professionals are uneducated on the topics of neurodiversity and ableism. Clinicians providing autism diagnoses often use pathologizing language and focus on negative aspects of autism (Crane et al., 2018; Dwyer et al., under review; Jegatheesan et al., 2010), and the discourse in autism intervention articles regrettably confirms that negative, subjective pathology language is widely employed in this field. However, guidance on alternative, more positive terminology is available (Bottema-Beutel, et al., 2020; Brown et al., 2021; Bury et al., 2020a; Kenny et al., 2016; Robison, 2019) and is currently gaining popularity among clinicians, researchers, and autism professionals. To align with this, we recommend that clinicians become immersed in the foundational essay of the Autistic advocacy movement that provided a plea to parents of Autistic people to accept their children as they are (Sinclair, 1993), while recognizing that this is not incongruent with working to improve core developmental and social competencies or maximize “person-environment fit” (Lai et al., 2020).

Importantly, acceptance of autism by others is correlated with lower levels of stress and depression in Autistic adults (Cage et al., 2018). Additionally, parental resolution and acceptance of children’s autism diagnoses, as well as parents’ tendency to take the perspective of their children, are associated with parental attunement to children in play interactions (Di Renzo et al., 2020). Therefore, it is important that professionals also support parents/caregivers in making intervention decisions that most align with the ideals of neurodiversity and the consensus of the Autistic community. Clinicians and professionals are encouraged to introduce parents to the neurodiversity approach, as well as direct attention towards the strengths of Autistic individuals and how these might contribute to future positive outcomes (see Bury et al., 2020b; Carter et al., 2015; Russell et al., 2019; Warren et al., 2020). Caregivers, particularly those of very young children, play a necessary part in the creation of child goals and shaping the program of behavioral intervention for their child. By introducing families to the neurodiversity perspective, autism professionals can empower caregivers to make these important decisions in a way that incorporates family values and culture while also focusing on the individuality and acceptance of their Autistic child. To facilitate this process, it is the responsibility of the professionals working with the families to provide access to neurodiversity-focused resources and information. More specifically, neurodiversity-informed pedagogy should be embedded into existing clinical programs to enhance the training of professionals and ensure they are capable of serving as the “neurodiversity advocate” when working with Autistic individuals and their families. Clinicians who are aware of these issues are in a position to provide a more balanced view that includes discussion of personal strengths and encourages acceptance. Because the focus of this paper is on NDBI interventionists, our suggestions are particularly relevant to Board Certified Behavior Analysts (BCBAs) and those working with them. However, these suggestions extend to professionals across the board, including psychologists, pediatricians, allied health professionals, and teachers. Neurodiversity-focused resources should be made available to families beginning in the diagnostic process (i.e. providing resources on diagnostic reports, discussing with families during diagnostic feedback sessions; see Brown et al., 2021) and throughout the intervention experience. A useful resource in its own right, the Autistic Self Advocacy Network’s recent publication *Start Here: A Guide For Parents of Autistic Kids* (2021) has a list of additional resources that can be shared with parents.

#### Centering Autistic Voices

Autistic perspectives in behavioral intervention planning and implementation efforts must be centered, especially given the NDBI emphasis on following the client’s lead within the natural environment. To meet this goal, we encourage behavioral intervention companies to seek out and confer with Autistic consultants to give feedback on client goals and program implementation. While the responsibility to educate others does not lie solely with the Autistic community, it can be helpful for providers to collaborate with and learn from those on the spectrum (cf. Fletcher-Watson et al., 2019). Increasing the number of Autistic partners and consultants in university settings (within clinical training programs), behavioral agencies, and other settings where intervention is provided is critical. Given the current divide between Autistic adults and interventionists, as well as other practical concerns, this may not always be a feasible approach for large-scale implementation. Therefore, it is also important that agencies and professionals take it upon themselves to seek out research and other resources (blogs, websites, etc.) that outline the perspectives and concerns of the Autistic community (e.g., Lichtlé et al., 2021).

Beyond hiring Autistic people into consultant and partner roles, it is also imperative for NDBI providers to ensure that the field is accessible and inclusive for Autistic people wishing to pursue professional careers delivering NDBIs. In fact, Autistic adults may be uniquely positioned to serve as intervention providers (e.g. Hillman et al., 2020) and educators on the topic of neurodiversity (Gillespie-Lynch et al., 2017), and thus it is arguably desirable to increase Autistic representation among behavior analysts. Some of the changes discussed here—such as avoiding the use of pathologizing language—might help to foster a culture that is more welcoming to Autistic NDBI professionals. In addition, any gatekeeping requirements that could pose barriers to Autistic professionals and trainees should be scrutinized and ameliorated. Characterization of such barriers, in addition to more general inquiry into the practice of Autistic BCBAs, could be an important direction for further research.

It is also important to note that a *variety* of voices must be taken into account. Autism research participants have historically been predominantly white male samples (Pierce et al., 2014; West et al., 2016). It will not be possible to understand the depth and complexity of all Autistic voices until we listen to those across the gender and ethnicity spectrum, previewed in the anthology, *All the Weight of Our Dreams: On Living Racialized Autism*, in which diverse Autistic individuals shared their perspectives and experiences (Brown et. al., 2017). In order to provide appropriate services to diverse Autistic individuals and their families, it is imperative that interventionists seek the input of Autistic people from a similar sociocultural background and identity group as their current clients.

#### Supporting an Autistic “Way of Being”

Incorporating Autistic voices in the development and refinement of intervention programs will ultimately lead to more socially valid interventions. With input from the most important stakeholders—Autistic individuals themselves—intervention goals, procedures, and outcomes are likely to be seen as more acceptable, useful, and effective (Wolf, 1978). However, it is important to note that intervention goals do not necessarily need to be *desirable/preferable* to young children themselves. Indeed, neurotypical children are encouraged to work on many important skills that they may not enjoy, for example cleaning up, waiting in line, and washing their hands. The key is that goals for Autistic children need not only be developmentally and functionally relevant, but also need to be congruent with the development of an Autistic “way of being” (Sinclair, 1993). For example, being able to successfully wait in line will allow children access to a variety of environments and activities, and teaching this skill does not appear to be incongruent with the neurodiversity paradigm. Hence, “learning to wait” may be considered a reasonable intervention goal. On the other hand, goals that focus on promoting socially desirable responses for the sake of others’ comfort must be reassessed. For example, teaching a young child to limit their talking about their own interest so as to seem like a better conversational partner may make social interactions appear smoother from a neurotypical point of view, but it may inadvertently encourage masking of the child’s Autistic way of being. Psychoeducation about how this and other similar social behaviors may be interpreted might be warranted with older children, taking care to highlight that Autistic individuals’ social skills are simply different, not worse than, neurotypicals’, and that this can lead to misunderstandings (known as the “double empathy problem”; Milton, 2012). However, behavioral goals of this nature for young children are likely to not be seen as socially valid. A helpful exercise for clinicians might be to imagine their clients as adults, reading through their childhood behavioral plans—would they find the goals offensive? Would they feel that their identity was being extinguished? Or would they feel that they were being given reasonable supports to learn valuable skills and navigate the world as an Autistic individual? Discussing intervention principles and goals with children (when developmentally appropriate) will help ensure intervention social validity.

Though clinicians can use Autistic perspectives to guide their practice, NDBI goal formation and implementation is a very family-centered process, and parents and other caregivers should have considerable input. Similar to clinicians and teachers, parents often have goals for their children that are not always in line with what the children find enjoyable. For example, parents might want their child to learn appropriate hygiene, such as taking baths, or to stop watching TV before bed, neither of which will always be pleasant tasks for young children of any neurotype. However, neither goal appears to be encouraging masking, and both are likely compatible with developing an Autistic way of being while simultaneously preparing the child for something they will encounter across their life. A parent’s goal for their child to always hug family members goodbye, on the other hand, is likely to be viewed negatively, as it ignores potential sensory issues associated with touch and emphasizes compliance over consent. Clinicians therefore must work with families to find goals that are congruent with both familial and cultural values (Wang et al., 2007) as well as the values of neurodiversity. This will require clinicians to provide information regarding goals that may or may not be congruent with neurodiversity as part of the parent training that is often part of NDBI programs. It is of course still necessary to establish that the *procedures* used to build the skill are also socially valid, though utilizing the motivational principles and natural reinforcement inherent in NDBIs is likely to result in socially valid procedures.

Though respecting an Autistic individual’s way of being is absolutely crucial, it may sometimes be difficult for clinicians to assess which intervention targets enhance quality of life. This is particularly true given that autism is diagnosed based on a set of behaviors, and while all are arguably part of autism, these behaviors might be positively or negatively associated with quality of life in ways that could depend on the contexts and environments surrounding Autistic individuals. For example, atypical or decreased use of gestures factors into the diagnostic algorithm for both the Autism Diagnostic Observation Scale (ADOS; Lord et al., 2012) and Autism Diagnostic Interview–Revised (ADI-R; Lord et al., 1994). It is therefore reasonable to say that non-neurotypical gesturing can be a part of the Autistic way of being. However, teaching a child to gesture in order to communicate their needs will likely improve their quality of life. Should teaching gestures therefore be an intervention goal? We argue, yes, it should be if—and only if—the purpose of the goal is to enhance the child’s ability to communicate (e.g. teaching signs for things the child likes, teaching pointing to indicate preference). If the intention behind the goal is instead to increase the social desirability of the child’s gesturing (e.g. by emphasizing waving hello/goodbye or insisting on increased use of descriptive gestures), then this is no longer an appropriate goal. (It should also be noted that teaching gestures such as pointing or other “neurotypical” forms of indicating preference or desire should not necessarily be prioritized over other communicative actions, such as using alternative and augmentative communication [AAC] devices, depending on the individual’s preferences and abilities.) Therefore, while basing intervention goals solely on ADOS and ADI-R criteria is never warranted, it is possible in some cases that working on goals that increase quality of life may coincide with lower ADOS and ADI-R scores. While these decreased scores may be associated with improved adaptive functioning, it does not imply a “reduction” in autism or a fundamental change in a person’s identity (see Tsatsanis et al., 2011 for a discussion on targeting adaptive behaviors as opposed to “symptomatology”). Working with Autistic individuals when creating intervention plans is one way to address this ambiguity.

#### Assessing Social and Ecological Validity

While much can be done to improve the social validity of interventions while they are being developed, researchers and clinicians must also do more to continually assess the social validity of the interventions they are using. This is particularly true given that the Autistic community is not a monolith, and an intervention’s social validity will vary from person to person. Social validity is especially relevant for the implementation of NDBIs, given their theoretical basis in the natural environment and following the child’s lead, and as such should be regularly incorporated into NDBI practice. We recommend that all NDBI research trials and clinical programs include methods for feedback and measures of social validity, such as the Scale of Treatment Perceptions (STP; Berger et al., 2016).

However, while parent and professional input is valuable, it is not enough to simply ask caregivers and clinicians if they thought an intervention was acceptable; it is imperative to include Autistic perspectives as well. We recommend that measures like the STP (Berger et al., 2016) be adapted into self-report versions that can be made available for Autistic clients. Furthermore, existing caregiver/professional social validity questionnaires can be modified to encourage these individuals to solicit Autistic input and ensure that outcomes of interest to Autistic people are captured. Because not all intervention participants can fill out a survey, new methods of assessing social validity and client satisfaction must also be developed. For example, individuals’ attendance, engagement, and emotional state before and during an intervention session could be tracked. Positive verbal statements, smiles, laughter, and spontaneous requests to continue could also be tracked as social validity data. Likewise, protests, avoidant or aggressive behavior, and tantrums could be interpreted as a lack of social validity (see Robinson, 2011, for an example of this kind of social validity check used in NDBI research). While some discomfort is expected when any young child is asked to do something they are not interested in, an unexpectedly and/or intensely negative response should always prompt clinicians to reevaluate the situation, instead of assuming that distress is warranted. Adequately monitoring these and other adverse events in clinical trials and clinical programs is yet another way to ensure an intervention is doing more good than harm (Bottema-Beutel et al., 2021a; Dawson & Fletcher-Watson, 2021). Tesfaye et al. (2019) also present alternative ways of capturing first-person perspectives in youth with disabilities, such as engaging in art and photography and sorting picture cards of activities into “like” versus “dislike” categories (also see Courchesne et al., 2021). This kind of “ethical listening” (that is, paying attention to what one is communicating through non-speaking means; Lebenhagen, 2019) is crucial to including the perspectives and preferences of *all* Autistic people.

While we argue that NDBIs can eventually be *theoretically* aligned with the goals of neurodiversity, the actual providers implementing NDBIs in the community determine if this aspirational objective is met. To this end, more research needs to be done to explore how NDBIs are translated from a tightly controlled research context into community-based clinical and educational settings (e.g., Stahmer et al., 2017, 2019; Waters et al., 2020). Conducting this research may be challenging, especially given that clinicians in the community have highly variable levels of education and knowledge of evidence-based practices (Stahmer et al., 2005). This gap in knowledge is reflected in a recent meta-analysis by Nahmias et al. (2019), which revealed smaller effect sizes in community early intervention programs compared to university-based programs. Future research must not only look at *outcomes* of community-based NDBI programs, but also assess the extent to which programs are being implemented in ways that preserve the values of naturalistic intervention approaches and follow the core tenets of neurodiversity (Stahmer & Pellecchia, 2015).

Special care must also be directed toward recruiting diverse families into NDBI research studies (Shaia et al., 2020). An Autistic client’s and family’s cultural background will inform their beliefs on what constitutes a disability, appropriate intervention goals, and acceptable avenues for intervention (de Leeuw et al., 2020; Ravindran & Myers, 2012). Exposure to and acceptance of neurodiversity is one such factor that may influence these beliefs. Indeed, given the existence of cross-cultural differences in attitudes and stigma towards autism and disability (de Vries et al., 2020; Gillespie-Lynch et al., 2019; Someki et al., 2018), interventionists may sometimes face the challenging task of respecting clients’ cultural beliefs while also encouraging caregivers to embrace a neurodiversity-aligned perspective. The first step in this process is for clinicians and researchers to develop their own cultural awareness and acknowledge their own personal biases (Fong et al., 2016). The use of assessments such as the Multicultural Sensitivity Scale (Jibaja et al., 1994) or the Diversity Self-Assessment (Montgomery, 2001) can facilitate the identification of any potential cultural barriers (such as modalities of communication or expression of emotions). Part of this cultural sensitivity is also recognizing that some individuals may see themselves as, in addition to many other things, part of an Autistic culture (Straus, 2013).

#### Participatory Research

An additional measure academics and service providers can take to ensure validity of NDBIs is to include Autistic individuals and their families in the process at all stages of clinical trial or clinical program development (Fletcher-Watson et al., 2019; Leadbitter et al., 2021; Milton, 2014; Nicolaidis et al., 2019). Though participatory action research comes with its own set of challenges (Fletcher-Watson et al., 2019; Nicolaidis et al., 2011), it is much easier to incorporate Autistic insights into an intervention program from its inception rather than to change existing practices. While many NDBIs are clearly past the inception phase, future intervention trials will benefit from including Autistic co-researchers and/or consultants at all stages of the research process from question generation to the dissemination of results (Nicolaidis et al., 2019). In addition, as discussed in preceding sections, further modification of NDBIs to align with a neurodiversity approach seems in order, and participatory research could be used to make these reforms.

#### Conflicts of Interest

Furthermore, the circumscribed conflict of interest reporting that has been commonplace in ABA research (Bottema-Beutel et al., 2021b) is clearly another domain in which this field must improve. It is imperative for NDBI researchers to disclose any relevant conflicts of interest, for example working in a clinical capacity at a facility where NDBIs are regularly provided or receiving royalties from the sale of an NDBI treatment manual (see this paper’s conflict of interest statement for further examples). Indeed, more broadly, profit motives and commodification in the autism intervention “industry” have been scrutinized and subjected to understandably harsh criticism (Broderick & Roscigno, 2021; Dawson, 2004). Beyond monetary gains, there is also the concern of experimenter allegiance, which has influenced the effect sizes and biased the outcomes of published clinical trials (Dragioti et al., 2015). It is possible that non-profit approaches to curriculum design and community implementation might offer less risk of bias, and moreover might be more readily trusted by community members with concerns about intervention, than for-profit models.

#### Adaptive Treatment Designs

Finally, recent intervention research models, such as the Sequential Multiple Assignment Randomized Trial (SMART; Collins et al., 2007), hold promise for systematizing a personalized approach to NDBIs (Kasari et al., 2018). For example, if children do not easily pick up spoken language after an initial period of intervention, an AAC device may be added (see Kasari et al., 2014 for an example of an NDBI used in conjunction with a speech generating device). It will be crucial for researchers and interventionists implementing SMART designs to consider a change in intervention not only if an individual is categorized as a treatment “non-responder,” but also if they experience adverse effects or show signs of not finding the intervention socially valid. Finally, NDBIs may also be aided if researchers consider adopting a subgroup perspective such that interventions are responsive to the heterogeneity of the autism spectrum (Kim et al., 2016; Uljarević et al., 2020).

## Conclusions

We readily acknowledge the conflict between the neurodiversity movement’s objective of securing autism acceptance and behavioral intervention’s past and present legacy of attempting to normalize Autistic individuals. However, in alignment with other researchers (Fletcher-Watson et al., 2018; Lai et al., 2020; Leadbitter et al., 2021), we believe that these perspectives are *not* incompatible and argue that naturalistic, strength-based approaches such as NDBIs have the potential to create a unifying framework to combine the goals of behavioral intervention proponents and neurodiversity advocates. While some will undoubtedly continue to feel that use of behavioral principles will never be appropriate for use with human subjects (e.g. Therapist Neurodiversity Collective, n.d.; Tolley, nd.), we believe that interventions that combine behavioral and developmental theory have the capability to work in concert with the core tenets of the neurodiversity movement. Such alignment of NDBIs and neurodiversity is far more likely in cases where clinicians and researchers partner with the Autistic community, seek out opportunities to improve their practices, and remain open to feedback and change.

NDBIs use a strength-based model focused on using child interests and motivation in order to teach skills that are useful and functional for them. Though not the only behavioral approach that attempts to address social validity, NDBIs present one useful reference point for those interested in reforming behavioral intervention. With continued planning and intentional commitment to reform, the practice of NDBIs can be shifted further away from the reduction of core Autistic traits and toward the improvement of well-being and optimization of individual potential. Furthermore, intervention goals should be individualized and based on a nuanced, balanced understanding of both Autistic and neurotypical development and behavior. We additionally call for clinicians and researchers to consult with Autistic individuals when designing intervention programs to ensure we are targeting meaningful goals and not causing distress or stifling Autistic ways of being. Furthermore, we encourage ongoing assessments of social and ecological validity, especially from Autistic points of view. It is simply not enough to show improvement on depersonalized, “objective” outcomes if participants’ voices and needs are ignored, especially if those outcomes conflict with personal objectives. More generally, we believe it is important for researchers to explore the opinions and feedback of members of the Autistic community, as well as those of the broader neurodiversity movement, regarding intervention goals and practices. Listening to what Autistic individuals think specifically about the acceptability of NDBIs will allow us to further identify areas in which these interventions are succeeding and where they should be improved. Including other, non-Autistic stakeholders as participants in this kind of research will also help uncover places where there are overlaps as well as divergences in perspectives. We believe that the solicitation of stakeholder opinions is a necessary step forward in reforming behavioral intervention, and the authors of this manuscript have begun several ongoing studies to investigate this topic further.

Finally, we encourage NDBI practitioners to also be transparent about their approach, its strengths and limitations, and their commitment to acknowledging neurologically diverse perspectives. Families face considerable difficulty simply learning about, advocating for, and selecting among available supports (Brewer, 2018; Tzanakaki et al., 2012), and when providers fail to provide families with the information they need to make informed choices, this task naturally becomes even more challenging. Thus, if professionals implement the recommended strategies to deliver interventions in a more neurodiversity-aligned manner, families should be informed and empowered to choose these neurodiversity-aligned interventions over other models. Indeed, this recommendation extends beyond NDBI practitioners to all diagnosticians, interventionists, educators, and disability service providers (see also Brown et al., 2021). It is vital that professionals in each of these communities become informed about neurodiversity and discuss it with the families with whom they work.

As a society, we must also consider how our physical spaces and perspectives still prevent meaningful inclusion (Bölte, 2019; Pellicano et al., 2018). When designing curriculum, planning intervention goals, or engaging in discourse, professionals must be mindful of the messaging and support they are offering for Autistic people (and what information about autism they are conveying to non-Autistic individuals). Although accommodations are required by law in many jurisdictions, they are frequently insufficient in providing meaningful participation (Mcguire et al., 2006). It is time for professionals to not only focus on facilitating change within each individual client, but also to serve as advocates and champions for the removal of systemic barriers and the promotion of neurodiversity.

## Footnotes

1 We use identity-first language to acknowledge the preference of the Autistic community (see Bury et al., 2020a; Kenny et al., 2016; these studies also indicated some preference for “on the autism spectrum,” which is used occasionally herein as well). We also capitalize “Autistic” to highlight that the word is a proper noun or adjective that refers to a particular Autistic community/identity that is shared by many (though not necessarily all) Autistic individuals (see Brown, 2013; Johnson, 2021).
